# Psychometric properties of the itch numeric rating scale, skin pain numeric rating scale, and atopic dermatitis sleep scale in adult patients with moderate-to-severe atopic dermatitis

**DOI:** 10.1186/s12955-021-01877-8

**Published:** 2021-10-23

**Authors:** Jonathan I. Silverberg, Amy DeLozier, Luna Sun, Jacob P. Thyssen, Brian Kim, Gil Yosipovitch, Fabio P. Nunes, P. Cristian Gugiu, Helen A. Doll, Lawrence F. Eichenfield

**Affiliations:** 1 George Washington University, Washington, DC USA; 2 Lilly Corporate Center, Eli Lilly and Company, Indianapolis, IN 46285 USA; 3 Bispebjerg Hospital, University of Copenhagen, Copenhagen, Denmark; 4 Pied Piper Consulting, LLC, St. Louis, MO USA; 5 Miller School of Medicine, University of Miami, Miami, FL USA; 6 Clinical Outcomes Solutions, Chicago, IL USA; 7 Clinical Outcomes Solutions, Folkestone, Kent UK; 8 University of California San Diego, San Diego, CA USA; 9 Rady Children’s Hospital, San Diego, CA USA

**Keywords:** Atopic dermatitis, Atopic dermatitis sleep scale, Convergent-divergent validity, Itch NRS, Numeric rating scale, Patient-reported outcome, Psychometric, Reliability, Responsiveness, Skin pain NRS, Validity

## Abstract

**Background:**

The Itch Numeric Rating Scale (NRS), Skin Pain NRS, and Atopic Dermatitis Sleep Scale (ADSS) are self-administered patient-reported outcome (PRO) instruments developed to assess symptoms in patients with atopic dermatitis (AD). The objective of this study was to evaluate the psychometric properties (reliability, validity, and responsiveness) and interpretability thresholds of these PROs using data from three pivotal Phase 3 studies in adults.

**Methods:**

BREEZE-AD1, BREEZE-AD2, and BREEZE-AD5 evaluated the safety and efficacy of baricitinib in adults with moderate-to-severe AD. Clinician-reported outcomes and other PROs commonly assessed in patients with AD were used to estimate meaningful changes and evaluate test–retest reliability, convergent and divergent validity, known-groups validity, responsiveness, and meaningful change thresholds (MCTs) of the Itch NRS, Skin Pain NRS, and ADSS.

**Results:**

The test–retest reliability of the Itch NRS, Skin Pain NRS, and ADSS was evidenced by generally large intraclass correlation coefficients (> 0.7) in stable groups of patients between baseline and Week 1 and Weeks 4 and 8. Moderate-to-large correlations (*r* > 0.4) at baseline and Week 16 were generally observed between each measure and other PROs measuring the same concept, supporting convergent validity. Small-to-moderate correlations with clinician-reported outcomes demonstrated divergent validity. Each instrument was able to distinguish between known groups of disease severity as assessed using other indicators of AD severity. The responsiveness of the Itch NRS, Skin Pain NRS, and ADSS scales was demonstrated through significant differences in their change scores from baseline to Week 16 between categories of change in another PRO also from baseline to Week 16. Thresholds for interpreting meaningful change were estimated as − 4.0 for the 0–10 Itch and Skin Pain NRS items; − 1.25 for the 0–4 ADSS Items 1 and 3 and; − 1.50 for the 0–29 ADSS Item 2, these equivalent to moderate degrees of change.

**Conclusions:**

Results of this study demonstrate that the psychometric properties of the Itch NRS, Skin Pain NRS, and ADSS are good to excellent. These findings support the use of these instruments in daily assessment of AD symptoms in adults with moderate-to-severe AD.

*Trial registration* ClinicalTrials.gov numbers: NCT03334396, NCT03334422, and NCT03435081.

## Background

Patients with moderate-to-severe atopic dermatitis (AD) experience a heavy disease burden that substantially impacts both physical and mental functioning. Intense itch, skin pain, and related sleep disturbance are highly prevalent symptoms that patients with AD report as significantly affecting their quality of life (QoL) [1, 2]. The most commonly used instruments to assess the severity of AD include the Investigator Global Assessment (IGA) and the Eczema Area and Severity Index (EASI) [3–5]. These instruments are based on a physician’s visual assessment of clinical signs, and thus fail to capture the patient-experienced symptoms of itch, skin pain, and their impact on sleep. Though itch, skin pain, and sleep disturbance are important to patients with AD, measurement of these burdensome symptoms in clinical trials has so far been limited. Specific patient-reported outcome (PRO) measures may be useful to understand the burden from these symptoms better.

The Itch Numeric Rating Scale (NRS), Skin Pain NRS, and Atopic Dermatitis Sleep Scale (ADSS) are PROs designed to specifically measure the severity of a patient’s itch and skin pain, and assess impact of itch on sleep, respectively. These tools were developed according to the Food and Drug Administration (FDA) PRO guidelines [6], as simple, self-administered assessments in daily electronic diaries used in AD clinical trials. Previous studies found that the Itch NRS, Skin Pain NRS [7], and ADSS had good content validity, i.e. represent aspects of disease that are meaningful to patients. However, the psychometric properties of each measure were not assessed. Instruments can assess clinically relevant information, but not have sufficient validity, reliability, or interpretability to be used in clinical trials or practice. These psychometric properties are needed to support the use of these measures in clinical trials. The objective of this study was to determine the reliability, validity, responsiveness, and meaningful change of the Itch NRS, Skin Pain NRS, and ADSS in patients with moderate-to-severe AD using data from three Phase 3 clinical trials.

## Methods

### Study population

BREEZE-AD1 (AD1), BREEZE-AD2 (AD2), and BREEZE-AD5 (AD5) were three multicenter, randomized, double-blind, placebo-controlled, parallel-group Phase 3 clinical trials that evaluated the safety and efficacy of once daily, oral baricitinib 1 mg, and 2 mg, and 4 mg (in AD1 and AD2 only) versus placebo in adult patients with moderate-to-severe AD. In each trial, patients were ≥ 18 years old and intolerant or inadequate responders to topical therapy. At screening and baseline, patients were required to have an EASI score ≥ 16, a validated Investigator Global Assessment for Atopic Dermatitis (vIGA-AD™) score ≥ 3, and a body surface area (BSA) involvement ≥ 10%. Full details of each study, including the primary efficacy and safety outcomes, have been reported previously [8, 9]. Each study was conducted with informed consent, under institutional review board approval, and in accordance with the Declaration of Helsinki (ClinicalTrials.gov numbers: NCT03334396 (AD1), NCT03334422 (AD2), and NCT03435081 (AD5)).

### Instruments used in the psychometric analyses

#### Itch NRS, Skin Pain NRS, ADSS

The Itch NRS is a single item designed to capture information on self-reported severity of worst itching each day. Patients were asked to rate itching severity based on the worst level of itching in the past 24 h using an 11-point scale from 0 (“no itch”) to 10 (“worst itch imaginable”). The single-item Skin Pain NRS assesses self-reported severity of worst skin pain each day. For this, patients were asked to select a number from 0 (“no pain”) to 10 (“worst pain imaginable”) that best described the worst level of skin pain in the past 24 h. The three-item ADSS captures self-reported impact of itch on sleep disturbance each day, including: difficulty falling asleep (Item 1); number of night-time awakenings (Item 2) and; difficulty falling back asleep after waking (Item 3) during the previous night. Each ADSS item was scored individually. For Items 1 and 3, patients were asked to select a score ranging from 0 (“not at all”) to 4 (“very difficult”). For Item 2, patients selected the number of times they woke up each night, ranging from 0 to 29 times. Patients only answered Item 3 if their answer to Item 2 was greater than 0. These three PROs were self-assessed using a daily electronic diary, starting at screening through Week 16. Information was entered into the electronic diary at the end of each patient’s day. For each measure, weekly mean scores using the previous 7 days were calculated if at least 4 of the 7 diary values were non-missing. Weekly averages were calculated at baseline (Week 0) and Weeks 1, 2, 4, 8, 12, and 16.

#### Other scales

The PROs used to evaluate the psychometric properties of the Itch NRS, Skin Pain NRS, and ADSS included: (1) the Dermatology Life Quality Index (DLQI) [10], a self-reported measure of the impact of AD on QoL; (2) the Patient Oriented Eczema Measure (POEM) (11), a self-assessed disease severity score; and (3) the Patient Global Impression of Severity-Atopic Dermatitis (PGI-S-AD). More specifically, the PGI-S-AD is a single item asking patients to rate their overall AD symptoms over the last 24 h, ranging from “no symptoms” to “severe.” The PGI-S-AD measure was collected in the daily diary along with the Itch NRS, Skin Pain NRS, and ADSS items; the other PROs (DLQI and POEM) were assessed during clinic visits. In addition, the clinician-completed EASI, an evaluation of disease extent and clinical signs, was used in the psychometric validation.

### Statistical analyses

The following psychometric evaluation methods used in this study are in accordance with the published FDA guidance for assessing the measurement properties of PROs [6] and recent psychometric consensus discussions and presentations [12]. Unless otherwise stated, all analyses were conducted on eligible patients from the intent-to-treat (ITT) population who had weekly mean scores for the Itch NRS, Skin Pain NRS, or ADSS items at baseline. Analysis at visits following baseline includes all patients who had data at baseline and at the respective follow-up days or visits. All analyses were conducted using SAS Version 9.3 or higher (SAS Version 9. 2013. Cary, NC, SAS Institute Inc.).

#### Test–retest reliability

Test–retest reliability, which measures if instrument scores are reproducible across time, was assessed in a stable patient population during the interval between Week 0 and Week 1 as well as between Weeks 4 and 8. Stable patients were defined as those in the ITT population with weekly mean PGI-S-AD scores between − 0.50 and + 0.50 during each time interval. Intra-class correlation coefficients (ICCs) were calculated between the initial and retest periods. An ICC of ≥ 0.70 was considered acceptable agreement [13–15].

#### Construct validity (convergent and divergent validity)

Construct validity refers to the degree to which scores from one measure are theoretically consistent with those of another measure. Convergent and divergent validity were assessed using Spearman’s correlations between each of the Itch NRS, Skin Pain NRS, and ADSS items, and the scores of the PGI-S-AD, DLQI, POEM, and EASI. All analyses were conducted at Weeks 0 and 16. The strength of correlations was interpreted using Cohen’s conventions, where > 0.70 is large, 0.40–0.70 is moderate, and < 0.40 is small [12–14, 16, 17].

It was hypothesized that convergent validity, evidenced by moderate or large correlations, would be demonstrated at Weeks 0 and 16 between each of the Itch NRS, Skin Pain NRS, and ADSS items with the other PROs related to AD symptoms (POEM, DLQI, and PGI-S-AD), and that divergent validity, evidenced by small-to-moderate correlations, would be demonstrated between each of the instruments of interest with the more distally related clinician-completed assessment (EASI).

#### Known-groups validity (discriminant validity)

Known-groups validity was assessed by exploring the ability of each instrument to discriminate between subgroups of patients with different underlying disease severity. Based on the evaluation of construct validity, measures correlating with the Itch NRS, Skin Pain NRS, or ADSS above the 0.35 criterion for acceptable correlations [18, 19] were considered in the analyses of known-groups validity.

Patients were stratified into severity groups based on baseline scores of PGI-S-AD (weekly mean score of < 3 “no symptoms to mild symptoms” and ≥ 3 “moderate-to-severe symptoms”) and POEM (scores 0–7 “clear to mild,” scores 8–16 “moderate-to-severe,” and scores 17–28 “severe to very severe” [11]. The weekly average scores on the Itch NRS, Skin Pain NRS, and ADSS items were assessed between these groups using independent samples *t*-tests (2 groups) and analysis of covariance (ANCOVA) controlling for the effects of age, race, and gender (> 2 groups). When ANCOVA was used, post hoc *t*-tests assessed the mean weekly score between consecutive severity groups. Any severity group with < 20 patients were omitted from the analysis to ensure sufficient data for interpretation.

#### Responsiveness

Responsiveness, the ability of the measure to detect change when change in the construct of relevance has occurred, was evaluated using ANCOVAs and post-hoc paired *t*-tests to assess significant differences in mean changes in the Itch NRS, Skin Pain NRS, and ADSS items from Week 0 to Week 4 and Week 0 to Week 16 between groups of patients with different degrees of change in the construct of relevance. The standardized response mean (SRM) [19] was used to interpret the magnitude of responsiveness of each measure; based on Cohen’s recommendations [19], SRMs of 0.20, 0.50, and 0.80 represent small, moderate, and large changes, respectively [20].

Mean changes were assessed within 4 change categories of the POEM: (1) “much improved” patients who moved more than one health category to a better health category (> 1 category improvement); (2) “improved” patients who moved by one health category to a better health category (1 category improvement); (3) “stable” patients who remained in the same health category (no category change); and (4) “declined” patients who moved to a worse health category (≥ 1 category worsening). These categories were based on changes from baseline to the respective time point in the POEM severity category (scores 0–7 “clear to mild,” scores 8–16 “moderate,” and scores 17–28 “severe to very severe” [11]. It was hypothesized that statistically significant differences in the Itch NRS, Skin Pain NRS, and ADSS items would be observed between POEM change categories [11]. Differences in change scores between groups were tested using ANCOVA, controlling for age, gender, and race [21]. Post hoc *t*-tests and SRMs between consecutive change groups were also conducted.

#### Meaningful change estimation

Meaningful change refers to the individual-patient level of differences in scores in the domain of relevance which patients perceive as meaningful [6].

##### Anchor-based assessment

An anchor-based analysis, with weekly mean PGI-S-AD serving as the anchor variable, was the primary method used to derive clinical interpretations of the Itch NRS, Skin Pain NRS, and ADSS items. Spearman’s correlations were evaluated between the PGI-S-AD weekly average score and each measure at baseline, Week 4, and Week 16. Spearman’s correlations were also used to compare the change in the PGI-S-AD weekly average with each measure’s weekly average from baseline to Week 4 and Week 16.

To determine within patient meaningful change thresholds (MCTs), patients were classified into response groups based on their level of change in the PGI-S-AD between baseline and Weeks 4 and 16. These groups included “very marked improvement” (≤ −2.5 weekly average score change), “marked improvement” (> −2.5 and ≤ −1.5), “minimal improvement” (> −1.5 and ≤ −0.5), “no change” (> −0.5 and < 0.5), “minimal worsening” (≥ 0.5 and < 1.5), and “marked worsening” (≥ 1.5). MCTs on the Itch NRS, Skin Pain NRS, and ADSS items were based on change from baseline to Week 16 (primary analysis) and baseline to Week 4 (sensitivity analysis) within PGI-S-AD severity groups. A range of MCT estimates (minimal, moderate, and large) were computed for changes in each measure based on observed changes in the minimal, marked, and very marked PGI-S-AD improvement groups. A final MCT estimate for each measure was taken as the MCT equivalent to a moderate degree of change.

##### Distribution-based methods

Meaningful change analyses were also supported by distribution-based methods, which identify the raw score change on a measure that will produce a prespecified effect size and which identify a change which is beyond measurement error [22]. Distribution-based estimates were derived using weekly averages of the Itch NRS, Skin Pain NRS, and ADSS items at baseline. MCT estimates equivalent to 0.2, 0.5, and 0.8 pooled SDs were calculated. The Standard Error of Measurement (SEM) was calculated using the ICC from the test–retest analysis.

#### Handling of missing data

For Weeks 1, 2, 4, 8, and 12, weekly mean scores for Itch NRS, Skin Pain NRS, and ADSS items were set to missing if there were fewer than 4 non-missing values in the 7-day period before the respective clinic visit. For Week 0 and Week 16 analyses, if there were fewer than 4 non-missing assessments during the week prior to the visit, the 7-day window was extended by 1 day at a time (up to a maximum of 7 additional days) until there were at least 4 non-missing values.

## Results

A total of 624 patients in AD1, 615 patients in AD2, and 440 patients in AD5 were included. Patients’ baseline demographics and scores for the instruments of interest and other assessments are listed in Table 1.

**Table 1:** Descriptive analysis of baseline demographic characteristics for BREEZE-AD1, BREEZE-AD2, and BREEZE-AD5

Characteristics	BREEZE-AD1 (N = 624)	BREEZE-AD2 (N = 615)	BREEZE-AD5 (N = 440)
Age, years	35.6 (12.81)	34.7 (12.77)	39.5 (16.06)
*Sex, n (%)*			
Male	391 (62.7)	381 (62.0)	224 (50.9)
Female	233 (37.3)	234 (38.0)	216 (49.1)
*Race, n (%)*			
White	366 (58.9)	421 (68.5)	251 (57.3)
African American	2 (0.3)	0 (0)	80 (18.3)
Asian	189 (30.4)	183 (29.8)	81 (18.5)
Other	64 (10.3)	11 (1.8)	26 (5.9)
*Alcohol use, n (%)*			
Never	214 (34.3)	220 (35.8)	103 (23.4)
Current	381 (61.1)	353 (57.4)	291 (66.1)
Former	29 (4.6)	42 (6.8)	46 (10.5)
*Tobacco use, n (%)*			
Never	394 (63.1)	389 (63.3)	276 (62.7)
Current	158 (25.3)	155 (25.2)	80 (18.2)
Former	72 (11.5)	71 (11.5)	84 (19.1)
Duration since AD diagnosis, years	25.7 (15.10)	24.2 (13.86)	23.6 (16.65)
Age at time of AD diagnosis, years	10.2 (14.54)	10.8 (14.22)	15.9 (20.30)
IGA of 3, n (%)	363 (58.2)	305 (49.7)	256 (58.2)
IGA of 4, n (%)	261 (41.8)	309 (50.3)	184 (41.8)
EASI	30.9 (12.45)	33.5 (13.44)	27.1 (11.29)
BSA	51.0 (22.34)	53.5 (22.62)	40.9 (22.73)
Itch NRS	6.5 (2.09)	6.6 (2.17)	7.2 (2.17)
Skin Pain NRS	5.8 (2.47)	6.05 (2.55)	6.56 (2.63)
ADSS Item 2	3.0 (4.67)	1.8 (2.31)	2.3 (2.89)
DLQI	13.6 (7.27)	14.4 (8.09)	14.7 (7.28)
PGI-S-AD	3.9 (0.82)	3.9 (0.85)	4.0 (0.85)
POEM	20.7 (5.59)	20.4 (6.26)	21.3 (5.62)

Data are mean (standard deviation) unless stated otherwise

AD, atopic dermatitis; ADSS, Atopic Dermatitis Sleep Scale; BSA, body surface area; DLQI, Dermatology Life Quality Index; EASI, Eczema Area and Severity Index; IGA, Investigator’s Global Assessment; N, number of total patients, NRS, numeric rating scale; PGI-S-AD, Patient Global Impression of Severity–Atopic Dermatitis; POEM, Patient Oriented Eczema Measure; PRO, patient-reported outcome

### Test–retest reliability

The results of the test–retest analysis for each instrument in each study are provided in Table 2. Across all studies, the ICCs ranged from 0.770 to 0.875 for the weekly average Itch NRS and from 0.753 to 0.845 for the weekly average Skin Pain NRS; this indicated acceptable agreement among stable patients using both 1-week and 4-week intervals. For ADSS Items 1, 2 and 3, the ICCs for the weekly average score ranged from 0.754 to 0.843, 0.585 to 0.921, and 0.671 to 0.784, respectively, indicating generally acceptable agreement using both 1- and 4-week assessment intervals. These high levels of agreement indicated that all measures had good test–retest validity.

**Table 2:** Test–retest reliability assessment of itch NRS, skin pain NRS and ADSS for BREEZE-AD1, BREEZE-AD2, and BREEZE-AD5

Measure	Study	Baseline to Week 1		Week 4 to Week 8	
		N	ICC^a^ (95% CI)	N	ICC^a^ (95% CI)
Itch NRS	BREEZE-AD1	620	0.863 (0.842, 0.882)	599	0.783 (0.750, 0.812)
	BREEZE-AD2	608	0.853 (0.830, 0.873)	587	0.816 (0.787, 0.841)
	BREEZE-AD5	432	0.875 (0.851, 0.896)	405	0.770 (0.727, 0.807)
Skin pain NRS	BREEZE-AD1	620	0.832 (0.806, 0.855)	599	0.773 (0.739, 0.804)
	BREEZE-AD2	608	0.845 (0.821, 0.866)	587	0.808 (0.778, 0.834)
	BREEZE-AD5	432	0.832 (0.801, 0.859)	405	0.753 (0.708, 0.792)
ADSS item 1	BREEZE-AD1	620	0.839 (0.814, 0.861)	599	0.754 (0.717, 0.786)
	BREEZE-AD2	607	0.843 (0.819, 0.865)	587	0.776 (0.742, 0.806)
	BREEZE-AD5	432	0.834 (0.803, 0.861)	405	0.792 (0.753, 0.826)
ADSS item 2	BREEZE-AD1	620	0.921 (0.908, 0.932)	599	0.903 (0.887, 0.917)
	BREEZE-AD2	607	0.765 (0.730, 0.796)	587	0.585 (0.529, 0.635)
	BREEZE-AD5	432	0.921 (0.906, 0.934)	405	0.918 (0.901, 0.932)
ADSS item 3	BREEZE-AD1	512	0.780 (0.743, 0.811)	353	0.703 (0.646, 0.752)
	BREEZE-AD2	497	0.761 (0.722, 0.796)	315	0.682 (0.618, 0.737)
	BREEZE-AD5	369	0.784 (0.741, 0.820)	239	0.671 (0.596, 0.735)

ADSS, Atopic Dermatitis Sleep Scale; CI, confidence interval; ICC, intra-class correlation coefficient; N, number of total patients, NRS, numeric rating scale

^a^An ICC of 0.70 and above is considered acceptable agreement

### Construct validity (convergent and divergent validity)

Results supporting convergent and divergent validity of the Itch NRS, Skin Pain NRS, and ADSS items are shown in Table 3. Moderate-to-large correlations between the reference PRO assessments of AD symptoms and the Itch NRS (*r* range: 0.483–0.762 at baseline and 0.586–0.834 at Week 16) and the Skin Pain NRS (*r* range: 0.474–0.727 at baseline and 0.549–0.768 at Week 16) supported convergent validity. Similarly, moderate correlations, supporting convergent validity, were generally observed between the PRO assessments and ADSS Item 1 (*r* range: 0.499–0.651 at baseline and 0.508–0.670 at Week 16), Item 2 (*r* range: 0.368–0.468 at baseline and 0.424 and 0.516 at Week 16), and Item 3 (*r* range: 0.403–0.639 at baseline and 0.466–0.809 at Week 16).

**Table 3:** Correlations between the Itch NRS, skin pain NRS, and ADSS with other instruments for BREEZE-AD1, BREEZE-AD2, and BREEZE-AD5 at baseline and week 16

Measure	Correlation	BREEZE-AD1^a^ correlation		BREEZE-AD2^a^ correlation		BREEZE-AD5^a^ correlation	
		Baseline	Week 16	Baseline	Week 16	Baseline	Week 16
Itch NRS	*ClinRO*						
	EASI	0.229	0.398	0.223	0.505	0.225	0.498
	*PRO*						
	PGI-S-AD	0.762	0.767	0.759	0.788	0.752	0.834
	DLQI	0.495	0.590	0.506	0.586	0.544	0.666
	POEM	0.483	0.607	0.586	0.640	0.505	0.667
Skin pain NRS	*ClinRO*						
	EASI	0.222	0.338	0.225	0.455	0.251	0.444
	*PRO*						
	PGI-S-AD	0.707	0.691	0.727	0.710	0.689	0.768
	DLQI	0.529	0.549	0.591	0.582	0.586	0.658
	POEM	0.474	0.575	0.634	0.621	0.536	0.657
ADSS item 1	*ClinRO*						
	EASI	0.281	0.363	0.140	0.403	0.233	0.376
	*PRO*						
	PGI-S-AD	0.651	0.582	0.624	0.597	0.627	0.670
	DLQI	0.570	0.576	0.598	0.554	0.522	0.608
	POEM	0.507	0.508	0.531	0.518	0.499	0.582
ADSS item 2	*ClinRO*						
	EASI	0.245	0.254	0.131	0.357	0.223	0.350
	PRO						
	PGI-S-AD	0.451	0.424	0.435	0.446	0.449	0.495
	DLQI	0.445	0.486	0.447	0.486	0.411	0.516
	POEM	0.368	0.436	0.468	0.489	0.422	0.473
ADSS item 3	*ClinRO*						
	EASI	0.298	0.237	0.152	0.394	0.187	0.300
	*PRO*						
	PGI-S-AD	0.555	0.636	0.603	0.641	0.639	0.809
	DLQI	0.516	0.466	0.526	0.505	0.516	0.581
	POEM	0.439	0.472	0.460	0.474	0.403	0.563

ADSS, Atopic Dermatitis Sleep Scale; ClinRO, clinician-reported outcome; DLQI, Dermatology Quality of Life Index; EASI, Eczema Area and Severity Index; NRS, Numeric Rating Scale; PGI-S-AD, Patient Global Impression of Severity–Atopic Dermatitis; POEM, Patient Oriented Eczema Measure; PRO, patient-reported outcome

^a^Spearman’s correlation coefficients were calculated as correlations between assessments and continuous reference measures. Concurrent validity was small if the resulting coefficient was < 0.4, moderate if the coefficient was > 0.4–0.7, and large if the coefficient was > 0.7

Small-to-moderate correlations, supporting divergent validity, were observed between the clinical assessment and the following: Itch NRS (*r* range: 0.223–0.229 at baseline and 0.398–0.505 at Week 16); Skin Pain NRS (*r* range: 0.222–0.251 at baseline and 0.338–0.455 at Week 16); ADSS Item 1 (*r* range 0.140–0.281 at baseline and 0.363–0.403 at Week 16); ADSS Item 2 (*r* range: 0.131–0.245 at baseline and 0.254–0.357 at Week 16), and; ADSS Item 3 (*r* range 0.152–0.298 at baseline and 0.237 and 0.394 at Week 16).

### Known-groups validity

Table 4 reports the findings of known-groups validity analysis of each instrument using PGI-S-AD and POEM subgroups to define AD severity. At baseline, in all 3 studies, compared with patients in the moderate categories, patients in the severe categories of the PGI-S-AD and POEM had significantly more itching (*p* < 0.0001), skin pain (*p* < 0.0001), sleep disturbance (*p* < 0.0001), night-time awakenings (*p* < 0.01), and difficulty falling back asleep after waking (*p* < 0.0001) as demonstrated by higher mean scores on Itch NRS, Skin Pain NRS, ADSS Items 1, 2, and 3, respectively. These findings suggest that the Itch NRS, Skin Pain NRS, and ADSS items are able to distinguish between known groups based on disease severity.

**Table 4:** Known-groups validity of the itch NRS, skin pain NRS, and ADSS using PGI-S-AD and POEM subgroups at baseline for BREEZE-AD1, BREEZE-AD2, and BREEZE-AD5

Measure		PGI-S-AD category		POEM category		
		≤ 3 (no to mild symptoms)	> 3 (moderate to severe symptoms)	0–7 (clear to mild)	8–16 (moderate to severe)	17–28 (severe to very severe)
Itch NRS	*BREEZE-AD1*					
	Sample size	118	497	13	125	470
	LSM (SE) at baseline	4.22 (0.296)	7.43 (0.254)	N/A^b^	5.35 (0.322)	7.46 (0.283)
	Between-group comparisons^a^	–	< 0.0001	–	N/A^b^	< 0.0001
	*BREEZE-AD2*					
	Sample size	110	490	26	126	445
	LSM (SE) at baseline	3.58 (0.502)	6.79 (0.471)	3.05 (0.628)	5.09 (0.537)	6.89 (0.510)
	Between-group comparisons^a^	–	< 0.0001	–	< 0.0001	< 0.0001
	*BREEZE-AD5*					
	Sample size	61	364	9	72	342
	LSM (SE) at baseline	3.95 (0.328)	7.59 (0.260)	N/A^b^	4.89 (0.362)	7.29 (0.286)
	Between-group comparisons^a^	–	< 0.0001	–	N/A^b^	< 0.0001
Skin pain NRS	*BREEZE-AD1*					
	Sample Size	118	497	13	125	470
	LSM (SE) at baseline	3.36 (0.372)	6.73 (0.319)	N/A^b^	4.58 (0.394)	6.75 (0.346)
	Between-group comparisons^a^	–	< 0.0001	–	N/A^b^	< 0.0001
	*BREEZE-AD2*					
	Sample size	110	490	26	126	445
	LSM (SE) at baseline	2.94 (0.617)	6.29 (0.580)	1.96 (0.723)	4.09 (0.619)	6.47 (0.587)
	Between-group comparisons^a^	–	< 0.0001	–	< 0.0001	< 0.0001
	*BREEZE-AD5*					
	Sample size	61	364	9	72	342
	LSM (SE) at baseline	3.55 (0.436)	7.07 (0.345)	N/A^b^	4.05 (0.446)	6.83 (0.353)
	Between-group comparisons^a^	–	< 0.0001	–	N/A^b^	< 0.0001
ADSS item 1	*BREEZE-AD1*					
	Sample size	118	497	13	125	470
	LSM (SE) at baseline	1.04 (0.167)	2.36 (0.143)	N/A^b^	1.45 (0.174)	2.38 (0.152)
	Between-group comparisons^a^	–	< 0.0001	–	N/A^b^	< 0.0001
	*BREEZE-AD2*					
	Sample size	110	490	26	126	445
	LSM (SE) at baseline	1.06 (0.286)	2.29 (0.269)	0.94 (0.341)	1.53 (0.292)	2.34 (0.277)
	Between-group comparisons^a^	–	< 0.0001	–	0.0089	< 0.0001
	*BREEZE-AD5*					
	Sample size	61	364	9	72	342
	LSM (SE) at baseline	1.04 (0.201)	2.54 (0.159)	N/A^b^	1.38 (0.209)	2.43 (0.165)
	Between-group comparisons^a^	–	< 0.0001	–	N/A^b^	< 0.0001
ADSS item 2	*BREEZE-AD1*					
	Sample size	118	497	13	125	470
	LSM (SE) at baseline	1.18 (0.809)	3.22 (0.694)	N/A^b	1.36 (0.805)	3.35 (0.706)
	Between-group comparisons^a^	–	< 0.0001	–	N/A^b^	0.0001
	*BREEZE-AD2*					
	Sample size	110	490	26	126	445
	LSM (SE) at baseline	1.00 (0.622)	2.11 (0.585)	0.91 (0.727)	1.46 (0.622)	2.15 (0.590)
	Between-group comparisons^a^	–	< 0.0001	–	0.2514	0.0024
	*BREEZE-AD5*					
	Sample Size	61	364	9	72	342
	LSM (SE) at baseline	1.12 (0.525)	2.87 (0.416)	N/A^b^	1.32 (0.529)	2.79 (0.418)
	Between-group comparisons^a^	–	< 0.0001	–	N/A^b^	0.0001
ADSS item 3	*BREEZE-AD1*					
	Sample Size	63	442	10	74	415
	LSM (SE) at baseline	1.42 (0.175)	2.46 (0.136)	N/A^b^	1.74 (0.172)	2.50 (0.139
	Between-group comparisons^a^	–	< 0.0001	–	N/A^b^	< 0.0001
	*BREEZE-AD2*					
	Sample size	60	423	11	84	385
	LSM (SE) at baseline	1.30 (0.267)	2.35 (0.240)	N/A^b^	1.63 (0.266)	2.38 (0.247)
	Between-group comparisons^a^	–	< 0.0001	–	N/A^b^	< 0.0001
	*BREEZE-AD5*					
	Sample size	38	324	4	48	309
	LSM (SE) at baseline	1.53 (0.190)	2.82 (0.137)	N/A^b^	2.02 (0.195)	2.72 (0.143)
	Between-group comparisons^a^	–	< 0.0001	-	N/A^b^	< 0.0001

ADSS, Atopic Dermatitis Sleep Scale, NRS, Numeric Rating Scale, PGI-S-AD, Patient Global Impression of Severity-Atopic Dermatitis, POEM0, Patient Oriented Eczema Measure; SD, standard deviation

^a^Between-group comparisons. The LS mean and SE are derived from an ANCOVA adjusting for age, sex, and race. The *p* value for the pairwise comparisons between consecutive severity groups is assessing differences in scores between groups

^b^Where numbers were < 20 in any severity group, this severity group was omitted from the analysis and the analysis was conducted on the remaining severity groups

### Responsiveness

The responsiveness of the Itch NRS, Skin Pain NRS, and ADSS items between Weeks 0 and 16 and between Weeks 0 and 4 are shown in Tables 5 and 6, respectively. In all three studies, the magnitude of improvement in each instrument increased with greater improvement in the POEM, supporting the ability of each measure to detect change in the construct of relevance where change has occurred. For the Itch NRS and Skin Pain NRS, in each study at Weeks 4 and 16, the “much improved” group statistically significantly differed from the “improved” group (*p* < 0.001 for Itch NRS, *p* < 0.05 for Skin Pain NRS), and the “improved” category statistically significantly differed from the “stable” group (*p* < 0.0001 for both). In each study, at Week 16, the scores of each ADSS item increased with each improvement category; however, not all comparisons between consecutive improvement categories were statistically significant (Table 5).

**Table 5:** Within group mean and median change scores for responsiveness of the itch NRS, skin pain NRS, and ADSS to change on the POEM between baseline and week 16 for BREEZE-AD1, BREEZE-AD2, and BREEZE-AD5

	POEM groups at week 16			
	Much improved (> 1 category improvement)	Improved (1 category improvement)	Stable (No category change)	Declined (≥ 1 category worsening)
**Itch NRS**				
*BREEZE-AD1*				
Sample Size	71	198	263	25
Mean (SD) change	− 4.64 (2.333)	− 3.03 (2.025)	− 1.42 (1.986)	0.10 (1.964)
Median change	− 4.86	− 3.14	− 1.14	− 0.12
Between-group comparisons^a^	–	< 0.0001	< 0.0001	0.0007
*BREEZE-AD2*				
Sample size	85	163	263	23
Mean (SD) at baseline	− 4.49 (2.162)	− 3.01 (2.183)	− 1.47 (2.166)	0.13 (2.602)
Median change	− 4.5	− 2.86	− 1.29	0.07
Between-group comparisons^a^	–	< 0.0001	< 0.0001	0.0042
*BREEZE-AD5*				
Sample size	49	104	176	8
Mean (SD) at baseline	− 5.39 (2.523)	− 3.59 (2.245)	− 1.46 (2.109)	− 0.01 (3.105)
Median change	− 5.43	− 3.54	− 1.31	− 0.21
Between-group comparisons^a^	–	< 0.0001	< 0.0001	0.1167
**Skin pain NRS**				
*BREEZE-AD1*				
Sample size	71	198	263	25
Mean (SD) change	− 4.49 (2.580)	− 2.82 (2.289)	− 1.34 (2.220)	0.53 (2.236)
Median change	− 4.52	− 2.79	− 1.00	0.00
Between-group comparisons^a^	–	< 0.0001	< 0.0001	0.0002
*BREEZE-AD2*				
Sample size	85	163	263	23
Mean (SD) at baseline	− 4.60 (2.396)	− 3.17 (2.309)	− 1.36 (2.395)	0.04 (2.594)
Median change	− 4.29	− 2.74	− 1.29	0.25
Between-group comparisons^a^	–	< 0.0001	< 0.0001	0.0203
*BREEZE-AD5*				
Sample size	49	104	176	8
Mean (SD) at baseline	− 4.80 (2.842)	− 3.60 (2.398)	− 1.48 (2.354)	0.21 (4.211)
Median change	− 4.86	− 3.57	− 1.21	− 0.38
Between-group comparisons^a^	–	0.0216	< 0.0001	0.0932
**ADSS item 1**				
*BREEZE-AD1*				
Sample size	71	198	263	25
Mean (SD) change	− 1.40 (1.045)	− 0.98 (0.904)	− 0.51 (0.977)	0.34 (1.011)
Median change	− 1.17	− 0.91	− 0.29	0.00
Between-group comparisons^a^	–	0.0022	< 0.0001	< 0.0001
*BREEZE-AD2*				
Sample size	85	163	263	23
Mean (SD) at baseline	− 1.45 (0.924)	− 1.12 (0.951)	− 0.38 (0.937)	0.10 (0.739)
Median change	− 1.43	− 1.00	− 0.29	0.00
Between-group comparisons^a^	–	0.0144	< 0.0001	0.0453
*BREEZE-AD5*				
Sample size	49	104	176	8
Mean (SD) at baseline	− 1.69 (1.245)	− 1.30 (0.956)	− 0.34 (0.842)	− 0.06 (0.973)
Median change	− 1.71	− 1.07	− 0.14	0.00
Between-group comparisons^a^	–	0.0386	< 0.0001	0.5370
**ADSS item 2**				
*BREEZE-AD1*				
Sample size	71	198	263	25
Mean (SD) change	− 2.47 (4.141)	− 1.60 (2.491)	− 1.05 (2.954)	− 0.04 (1.483)
Median change	− 1.14	− 1.00	− 0.57	0.00
Between-group comparisons^a^	–	0.0431	0.0695	0.0957
*BREEZE-AD2*				
Sample size	85	163	263	23
Mean (SD) at baseline	− 1.63 (1.604)	− 1.36 (2.434)	− 0.70 (1.655)	− 0.43 (1.183)
Median change	− 1.14	− 0.86	− 0.45	− 0.29
Between-group comparisons^a^	–	0.2260	0.0004	0.6256
*BREEZE-AD5*				
Sample size	49	104	176	8
Mean (SD) at baseline	− 2.16 (1.665)	− 1.55 (2.631)	− 0.51 (1.526)	0.24 (0.592)
Median change	− 1.86	− 0.86	− 0.43	0.00
Between-group comparisons^a^	–	0.0753	0.0001	0.2183
**ADSS item 3**				
*BREEZE-AD1*				
Sample size	22	71	162	10
Mean (SD) change	− 1.00 (0.916)	− 0.77 (0.981)	− 0.32 (0.868)	0.70 (1.023)
Median change	− 0.98	− 0.71	− 0.23	0.65
Between-group comparisons^a^	–	0.2873	0.0011	0.0005
*BREEZE-AD2*				
Sample size	9	51	141	10
Mean (SD) at baseline	− 1.11 (0.906)	− 0.76 (0.869)	− 0.22 (0.940)	0.43 (0.838)
Median change	− 1.25	− 0.80	− 0.17	0.50
Between-group comparisons^a^	–	0.3592	0.0003	0.0574
*BREEZE-AD5*				
Sample size	15	52	115	2
Mean (SD) at baseline	− 1.60 (0.951)	− 1.27 (1.021)	− 0.18 (0.794)	− 0.79 (1.111)
Median change	− 1.86	− 1.14	0.00	− 0.79
Between-group comparisons^a^	–	0.4839	< 0.0001	0.2654

ADSS, Atopic Dermatitis Sleep Scale, ANOVA, analysis of variance; NRS, Numeric Rating Scale; POEM, Patient Oriented Eczema Measure; SD, standard deviation

^a^Between-group comparisons. The *p* value for the pairwise comparisons between consecutive severity groups was derived from an ANOVA assessing differences in score change between groups

**Table 6:** Within group mean and median change scores for responsiveness of the itch NRS, skin pain NRS, and ADSS to change on the POEM between baseline and week 4 for BREEZE-AD1, BREEZE-AD2, and BREEZE-AD5

	POEM groups at week 4			
	Much Improved (> 1 Category Improvement)	Improved (1 Category Improvement)	Stable (No Category Change)	Declined (≥ 1 Category Worsening)
**Itch NRS**				
*BREEZE-AD1*				
Sample size	63	191	301	26
Mean (SD) change	− 3.85 (1.943)	− 2.29 (1.779)	− 1.07 (1.629)	0.21 (1.942)
Median change	− 3.67	− 2.14	− 0.86	0.64
Between-group comparisons^a^	–	< 0.0001	< 0.0001	0.0002
*BREEZE-AD2*				
Sample size	69	190	286	23
Mean (SD) at baseline	− 3.90 (2.318)	− 2.45 (2.101)	− 1.08 (1.634)	0.14 (1.049)
Median change	− 3.71	− 2.29	− 0.86	0.17
Between-group comparisons^a^	–	< 0.0001	< 0.0001	0.0030
*BREEZE-AD5*				
Sample size	35	114	217	13
Mean (SD) at baseline	− 4.12 (2.261)	− 2.56 (2.059)	− 1.15 (1.627)	− 0.81 (1.569)
Median change	− 3.71	− 2.30	− 0.86	− 0.57
Between-group comparisons^a^	–	0.0002	< 0.0001	0.9320
**Skin pain NRS**				
*BREEZE-AD1*				
Sample size	63	191	301	26
Mean (SD) change	− 3.49 (2.111)	− 2.24 (1.970)	− 0.97 (1.773)	0.31 (1.726)
Median change	− 3.14	− 1.86	− 0.71	0.48
Between-group comparisons^a^	–	< 0.0001	< 0.0001	0.0007
*BREEZE-AD2*				
Sample size	69	190	286	23
Mean (SD) at baseline	− 4.13 (2.412)	− 2.66 (2.290)	− 0.97 (1.826)	0.20 (1.206)
Median change	− 4.00	− 2.29	− 0.86	0.43
Between-group comparisons^a^	–	< 0.0001	< 0.0001	0.0118
*BREEZE-AD5*				
Sample size	35	114	217	13
Mean (SD) at baseline	− 4.24 (2.361)	− 2.49 (2.119)	− 1.19 (1.889)	− 0.38 (1.667)
Median change	− 4.00	− 2.15	− 0.90	− 0.57
Between-group comparisons^a^	–	< 0.0001	< 0.0001	0.3379
**ADSS item 1**				
*BREEZE-AD1*				
Sample size	63	191	301	26
Mean (SD) change	− 1.31 (0.958)	− 0.75 (0.828)	− 0.42 (0.813)	0.31 (1.061)
Median change	− 1.29	− 0.71	− 0.29	0.00
Between-group comparisons^a^	–	< 0.0001	< 0.0001	< 0.0001
*BREEZE-AD2*				
Sample size	69	190	286	23
Mean (SD) at baseline	− 1.30 (0.886)	− 0.85 (0.894)	− 0.35 (0.823)	0.20 (0.507)
Median change	− 1.14	− 0.71	− 0.31	0.14
Between-group comparisons^a^	–	0.0001	< 0.0001	0.0062
*BREEZE-AD5*				
Sample size	35	114	217	13
Mean (SD) at baseline	− 1.49 (0.918)	− 0.88 (0.839)	− 0.46 (0.814)	− 0.12 (0.787)
Median change	− 1.43	− 0.88	− 0.29	0.00
Between-group comparisons^a^	–	0.0014	< 0.0001	0.2903
**ADSS item 2**				
*BREEZE-AD1*				
Sample size	63	191	301	26
Mean (SD) change	− 1.85 (2.836)	− 1.45 (3.027)	− 0.70 (2.119)	0.35 (2.133)
Median change	− 1.00	− 0.71	− 0.43	0.15
Between-group comparisons^a^	–	0.2945	0.0019	0.0375
*BREEZE-AD2*				
Sample size	69	190	286	23
Mean (SD) at baseline	− 1.34 (1.490)	− 1.12 (2.629)	− 0.52 (2.105)	0.12 (0.601)
Median change	− 0.86	− 0.56	− 0.43	0.00
Between-group comparisons^a^	–	0.3931	0.0096	0.1092
*BREEZE-AD5*				
Sample size	35	114	217	13
Mean (SD) at baseline	− 1.66 (1.522)	− 1.13 (1.365)	− 0.54 (1.208)	0.06 (0.791)
Median change	− 1.29	− 0.67	− 0.31	0.29
Between-group comparisons^a^	–	0.1450	0.0001	0.1491
**ADSS item 3**				
*BREEZE-AD1*				
Sample size	20	80	201	9
Mean (SD) change	− 1.15 (1.024)	− 0.73 (0.833)	− 0.31 (0.807)	0.27 (0.843)
Median change	− 1.21	− 0.66	− 0.20	0.29
Between-group comparisons^a^	–	0.0468	0.0002	0.0500
*BREEZE-AD2*				
Sample size	19	72	165	13
Mean (SD) at baseline	− 1.27 (0.700)	− 0.70 (0.814)	− 0.24 (0.849)	0.05 (0.472)
Median change	− 1.26	− 0.60	− 0.29	0.00
Between-group comparisons^a^	–	0.0069	< 0.0001	0.2670
*BREEZE-AD5*				
Sample size	14	48	138	7
Mean (SD) at baseline	− 1.54 (0.856)	− 1.05 (0.739)	− 0.41 (0.749)	− 0.47 (1.075)
Median change	− 1.38	− 1.00	− 0.36	− 0.29
Between-group comparisons^a^	–	0.1086	< 0.0001	0.6526

ADSS, Atopic dermatitis sleep scale, ANOVA, analysis of variance; NRS, numeric rating scale; POEM, patient oriented eczema measure; SD, standard deviation

^a^Between-group comparisons. The *p* value for the pairwise comparisons between consecutive severity groups was derived from an ANOVA assessing differences in score change between groups

### Meaningful change estimation

#### Anchor-based

Anchor-based estimates of the MCTs (minimal, moderate, and large) for each measure are listed in Table 7. For the 0–10 Itch NRS, the final estimate of meaningful change was − 4.0, with a reduction of 4 categories on the instrument consistent with moderate degree of change. Similarly, the final MCT for the 0–10 Skin Pain NRS was taken as − 4.0, also equivalent to a moderate degree of change. The final MCTs for ADSS Items 1, 2, and 3, respectively were − 1.25, − 1.50, and − 1.25, indicating that the smallest weekly averages are consistent with at least a moderate degree of improvement.

**Table 7:** Anchor-based estimates of MCTs for the itch NRS, skin pain NRS, and ADSS items in BREEZE-AD1, BREEZE-AD2, and BREEZE-AD5

Estimate (%)	Scale	BREEZE-AD1			BREEZE-AD2			BREEZE-AD5			Overall			Final MCT
Measure		MCT min	MCT mod	MCT large	MCT min	MCT mod	MCT large	MCT min	MCT mod	MCT large	MCT min	MCT mod	MCT large	
Itch NRS	0–10	− 2.25	− 4.00	− 6.00	− 2.25	− 4.50	− 6.00	− 2.50	− 4.50	− 7.00	− 2.25	− 4.25	− 6.50	− 4.00
Skin Pain NRS	0–10	− 2.00	− 4.00	− 6.00	− 2.25	− 4.50	− 6.00	− 3.00	− 4.00	− 7.00	− 2.25	− 4.00	− 6.50	− 4.00
ADSS Item 1	0–4	− 0.75	− 1.25	− 2.00	− 0.75	− 1.25	− 1.75	− 0.75	− 1.50	− 2.25	− 0.75	− 1.25	− 2.00	− 1.25
ADSS Item 2	0–29	− 1.00	− 1.75	− 3.00	− 0.75	− 1.50	− 2.00	− 1.00	− 1.50	− 3.00	− 1.00	− 1.50	− 3.00	− 1.50
ADSS Item 3	0–4	− 0.50	− 1.25	− 2.00	− 0.50	− 1.25	− 1.50	− 1.00	− 1.75	− 2.50	− 0.75	− 1.25	− 2.00	− 1.25

ADSS, Atopic Dermatitis Sleep Scale; MCT, meaningful change threshold; min, minimum; mod, moderate, NRS, numeric rating scale

#### Distribution-based

Distribution-based MCTs are listed in Table 8. Compared with anchor-based thresholds, SD and SEM estimates were smaller for all measures but the ADSS Item 2; this indicated that the anchor-based estimates are generally above measurement error and thus that improvements in these measures reflect a true improvement in condition severity. The larger distribution-based estimates for ADSS Item 2 reflected the large variability and skewness of this measure at baseline.

**Table 8:** Distribution-based estimates of MCTs for the Itch NRS, Skin Pain NRS, and ADSS items in BREEZE-AD1, BREEZE-AD2, and BREEZE-AD5

Measure	Study	Distribution-based estimates			
		0.2 SD	0.5 SD	0.8 SD	SEM
Itch NRS (scale 0–10)	BREEZE-AD1	0.42	1.04	1.67	0.88
	BREEZE-AD2	0.43	1.09	1.74	0.89
	BREEZE-AD5	0.43	1.09	1.74	0.92
	Overall	0.43	1.07	1.72	0.89
Skin pain NRS (scale 0–10)	BREEZE-AD1	0.49	1.23	1.98	1.09
	BREEZE-AD2	0.51	1.27	2.04	1.06
	BREEZE-AD5	0.53	1.32	2.11	1.20
	Overall	0.51	1.27	2.04	1.12
ADSS item 1 (scale 0–4)	BREEZE-AD1	0.22	0.54	0.86	0.49
	BREEZE-AD2	0.22	0.56	0.90	0.49
	BREEZE-AD5	0.24	0.60	0.95	0.52
	Overall	0.23	0.57	0.90	0.50
ADSS item 2 (scale 0–29)	BREEZE-AD1	0.93	2.34	3.74	1.39
	BREEZE-AD2	0.46	1.15	1.85	1.31
	BREEZE-AD5	0.58	1.45	2.32	0.82
	Overall	0.66	1.65	2.64	1.17
ADSS item 3 (scale 0–4)	BREEZE-AD1	0.19	0.47	0.76	0.48
	BREEZE-AD2	0.19	0.49	0.78	0.52
	BREEZE-AD5	0.19	0.47	0.76	0.51
	Overall	0.19	0.48	0.77	0.50

ADSS, Atopic Dermatitis Sleep Scale; MCT, meaningful change threshold; min, minimum; mod, moderate, NRS, numeric rating scale

## Discussion

This study evaluated the psychometric properties of the Itch NRS, Skin Pain NRS, and ADSS using data from three clinical trials of patients with moderate-to-severe AD. For each measure, assessment of test–retest reliability found high levels of agreement in stable groups of patients across all three studies for both 1-week and 4-week comparisons, indicating reliability of each instrument when no change would be expected. As hypothesized, the construct validity of each measure was also demonstrated, with moderate-to-large correlations with other PROs (POEM, DLQI, and PGI-S-AD) supporting convergent validity and smaller correlations with the more distally-related provider assessment (EASI) supporting divergent validity. These findings suggest that the Itch NRS, Skin Pain NRS and ADSS measure the underlying concept of AD symptomatology and, moreover, encapsulate unique information regarding disease symptoms, which can complement clinician-reported assessments in clinical trials. In addition, comparisons of the Itch NRS, Skin Pain NRS, and each ADSS item between PGI-S-AD and POEM severity categories demonstrated each measure’s ability to distinguish between known groups based on disease severity. Responsiveness was established through the ability of each instrument to discriminate significantly between subgroups of patients based on four change categories of the POEM (“much improved,” “improved,” “stable” and “declined”). Overall, the Itch NRS, Skin Pain NRS, and ADSS were determined to be highly reliable, valid, and responsive, supporting the use of these PRO instruments in daily assessment of AD symptoms in adults with moderate-to-severe AD.

Using anchor- and distribution-based analyses, thresholds for interpreting change of each measure were derived as criteria to assess treatment benefits in patients with AD. Four-point changes in the Itch NRS and Skin Pain NRS were found to demonstrate clinically meaningful responses in itch and skin pain severity, respectively. This 4-point change in the Itch NRS is consistent with minimal clinically important differences reported for similar itch scales [23, 24]. Changes of 1.25 points in ADSS Items 1 and 3 and 1.5 points in ADSS Item 2 were found to optimally demonstrate clinically meaningful improvements in sleep disturbance. These findings further confirm previous psychometric validation data of itch NRS in AD and psoriasis [23, 24].

The potential importance of these measures in clinical practice is indicated by the fact that patients with AD have identified itch, skin pain, and sleep disturbance as bothersome and distressing symptoms of their disease [25], but these are difficult or impossible for clinicians to assess using conventional tools. There is thus an unmet need for measures which can assess these patient-perceived symptoms. For example, EASI or BSA instruments assess important signs of disease, but these do not capture the impacts of itch, skin pain and sleep disturbance from AD as perceived by patients. Existing PROs of AD, such as the POEM, and Scoring Atopic Dermatitis or SCORAD include sleep items, but these items are included as part of a total score and do not assess the full impact of itch on sleep disturbance [11, 26]. These existing instruments are thus limited in their ability to accurately evaluate the impact of treatments on specific patient-reported symptoms in clinical trials. The implementation of the Itch NRS, Skin Pain NRS, and ADSS in AD clinical trials may therefore address this unmet need. Further, given the increasing use of electronic diaries in clinical settings, these low burden, simple, and specific PRO measures of symptoms may be useful in guiding treatment decisions in practice.

Though this study demonstrated strong evidence for the reliability, validity, and responsiveness of the Itch NRS, Skin Pain NRS, and ADSS, the data used in this psychometric validation are from a clinical trial and hence may not be generalizable to clinical practice. In addition, the inclusion and exclusion criteria of the three underlying studies limit this validation to adult patients with moderate-to-severe AD. Only a few patients were available in the mild group for assessing known-groups validity of each instrument using PGI-S-AD and POEM subgroups to define AD severity. The results of this study are also limited to a subset of patients who fluently spoke a language into which the assessment tool had been translated. The FDA recommends daily assessment of symptoms by patients as a shorter recall period allows for more reliable interpretation of symptom data [6]. However, while averaging scores over a 7-day period accounts for day-to-day variation in this analysis, this reduced variability may artificially increase the correlations with other measures [24]. Additionally, a similar study of itch severity measurement suggested a 7-day recall may be more clinically relevant [27]. Nevertheless, future studies are warranted to assess correlations between the Itch NRS, Skin Pain NRS and ADSS, which may further support the use of the three separate instruments in clinical practice.

## Conclusions

The results of this study demonstrate that the Itch NRS, Skin Pain NRS, and ADSS are highly reliable, valid, and responsive measures of symptoms that are important to patients with AD. In addition, each PRO is able to measure clinically important symptom changes in these patients. These findings support the use of these PRO instruments in clinical trials of patients with moderate-to-severe AD.

## Data Availability

The datasets generated and/or analyzed during the current study are not publicly available due to individual data privacy but may be available from the corresponding author on reasonable request. Use of the three measures can be requested from copyright@lilly.com.

## Abbreviations

AD: Atopic dermatitis
ADSS: Atopic dermatitis sleep scale
ANCOVA: Analysis of covariant
ANOVA: Analysis of variance
BSA: Body surface area
DLQI: Dermatology life quality index
EASI: Eczema area and severity index
FDA: Food and drug administration
ICC: Intra-class correlation coefficients
IGA: Investigator global assessment
ITT: Intent-to-treat
MCT: Meaningful change thresholds
NRS: Numeric rating scale
PGI-S-AD: Patient global impression of severity–atopic dermatitis
POEM: Patient oriented eczema measure
PRO: Patient-reported outcome
QoL: Quality of life
SEM: Standard error measurement
SD: Standard deviation
SRM: Standardized response mean
vIGA-AD: Validated investigator global assessment for atopic dermatitis
